# Oral and Topical Insecticide Response Bioassays and Associated Statistical Analyses Used Commonly in Veterinary and Medical Entomology

**DOI:** 10.1093/jisesa/ieaa041

**Published:** 2020-11-02

**Authors:** Edwin R Burgess, Bethia H King, Christopher J Geden

**Affiliations:** 1 Department of Biological Sciences, Northern Illinois University, DeKalb, IL; 2 USDA, ARS, Center for Medical, Agricultural, and Veterinary Entomology, Gainesville, FL

**Keywords:** toxicology, R programming, probit analysis, pesticide, house fly

## Abstract

Veterinary and medical entomologists who are involved in research on pest control often need to perform dose–response bioassays and analyze the results. This article is meant as a beginner’s guide for doing this and includes instructions for using the free program R for the analyses. The bioassays and analyses are described using previously unpublished data from bioassays on house flies, *Musca domestica* Linnaeus (Diptera: Muscidae), but can be used on a wide range of pest species. Flies were exposed topically to beta-cyfluthrin, a pyrethroid, or exposed to spinosad or spinetoram in sugar to encourage consumption. LD_50_ values for beta-cyfluthrin in a susceptible strain were similar regardless of whether mortality was assessed at 24 or 48 h, consistent with it being a relatively quick-acting insecticide. Based on LC_50_ values, spinetoram was about twice as toxic as spinosad in a susceptible strain, suggesting a benefit to formulating spinetoram for house fly control, although spinetoram was no more toxic than spinosad for a pyrethroid-resistant strain. Results were consistent with previous reports of spinosad exhibiting little cross-resistance. For both spinosad and spinetoram, LC_50_ values were not greatly different between the pyrethroid-resistant strain and the susceptible strain.

Dose–response bioassays can be used to assess the quantal response of any biological system to any stimulus (Yu 2015). Quantal means existing in one of two alternative states, e.g., dead or alive. The question being asked is whether the response is dose dependent, i.e., as dose increases, how does the proportion of individuals in each state change. Such bioassays usually involve assigning different magnitudes of a stimulus, e.g., doses of a chemical, to each group of the same biological system, followed by observation of the number of individuals per group that responded at a predetermined time endpoint, e.g., proportion dead at 48 h. This type of bioassay is widely used in drug and pesticide discovery and formulation. Medical and veterinary pests, such as filth flies, mosquitoes, and ticks, are often subjects of chemical susceptibility studies, for the purposes of both product development and resistance management (Kaufman et al. 2006, Pridgeon et al. 2008, Castro-Janer et al. 2010, Jiang et al. 2017). Thus, an insecticide research and development company might use this bioassay to compare a new insecticidal chemical to ones used previously. A researcher might also use a dose–response bioassay to compare the insecticide resistances of strains of the same species but with different exposure histories.

The present article is intended particularly for researchers new to running and/or analyzing such bioassays, a simple starter’s guide, as well as for researchers wanting to use the free program R for such analyses (R Core Team 2019) and having minimal experience with the program. Our R code (Supp Material [online only]) provides a simple, expedient way to generate all the necessary numbers for reporting typical dose–response bioassay results in peer-reviewed journals and technical documents. We highlight some key attributes of the assays and analyses and the reasons for them and provide a level of detail and step-by-step process that is not given in published papers. We do this using previously unpublished data from bioassays of house flies, *Musca domestica* Linnaeus (Diptera: Muscidae). However, the assays and analyses work well with little modification against a wide range of medical and veterinary pests, e.g., other filth flies, mosquitoes, and ticks. For species that require special dose–response protocols, the analyses and R code remain valid.

First, we describe an experiment in which house flies received a topical application of insecticide, and we describe how to determine the LD_50_. LD_50_ is the lethal dose of insecticide that kills 50% of a sample population in a given time period. Any value of LD can be determined from the described analysis (e.g., LD_1–99_), but we describe the importance of the LD_50_. Then we describe an experiment in which house flies were given insecticide impregnated in a sugar cube to encourage feeding on the insecticide, i.e., an oral experiment, and we describe how to determine the LC_50_. An LC_50_ is the lethal concentration that will kill 50% of a sample population within a given time period. The terms lethal dose (LD) and lethal concentration (LC) should not be used interchangeably when presenting results. An LD is defined as an exact amount of insecticide applied directly to an organism, e.g., units of insecticide mass applied per insect (e.g., ng/fly) or insecticide mass applied per average mass of insect tested (e.g., ng/mg of fly). For LC, insects are exposed to, or allowed to interact with, some amount of insecticide per period of time, but how much they actually make contact with or ingest is unknown, e.g., units of insecticide mass per area (e.g., mg/cm^2^), or per volume for aquatic insects, or in our LC assay here, the quantity of insecticide applied per mass of sugar (e.g., µg/g of sugar). Despite this distinction between dose and concentration, the term ‘dose response’, e.g., ‘dose–response bioassay’ and ‘dose–response curve’, is used even when determining LC.

Reasons that a 50% response (LD_50_ or LC_50_) is frequently used include 1) the 95% confidence interval (95% CI), occasionally called 95% fiducial limits, is narrowest at the 50% mortality mark. The 95% CI represents a range of values that we are 95% certain contains the true median dose or concentration that 50% of the entire population (vs just population sample) would respond to, e.g., would die from. CIs allow insecticidal treatments to be compared statistically (see Analyzing Data). 2) Typical analyses of dose–response curves, such as the analyses described here, assume a sigmoidal curve; with a sigmoidal curve, the 50% mortality mark is where the line is steepest, i.e., where a small change in dose or concentration causes a bigger change in mortality. Not as many individuals need to be tested to detect a big change in mortality, e.g., with just 10 individuals a difference of 49% versus 50% mortality will be hard to detect because the expected deaths are 5 for both, whereas a difference of 20% versus 50% mortality will be easier to detect because the expected deaths are 2 and 5, respectively.

The LD_50_ or LC_50_ and the 95% CI from dose–response bioassays are generated with probit analysis (Bliss 1934, Finney 1971). Probit analysis allows the researcher to make use of the sigmoidal nature of the response data, log-transforming the sigmoid response curve to a straight line and then performing a linear regression. The linear regression equation has the percent mortalities converted to probits, or ‘probability units’, on the y-axis and the logarithm of dose or concentration on the x-axis.

## Beta-Cyfluthrin and Spinosyns against House Flies

Our experiment with topical application used beta-cyfluthrin, a pyrethroid, and compared LD_50_ values at 24 h versus 48 h, using a pyrethroid-resistant strain of house fly. Our oral experiment examined LC_50_ values at 24 h and compared spinosad and spinetoram for a susceptible strain versus a pyrethroid-resistant strain.

House flies and other filth flies breed in manure and other decaying organic matter, often becoming pests to nearby livestock and humans (Gerry 2018). A wide range of insecticides are used for control. The pyrethroid beta-cyfluthrin is currently formulated as a premise spray for livestock facilities (SC Ultra Tempo by Bayer), where it is used as a broad-spectrum insecticide, including against flies. House fly populations exhibit varying degrees of resistance to beta-cyfluthrin (Kaufman et al. 2010, Scott et al. 2013, Khan et al. 2017).

Spinosad and spinetoram are broad-spectrum insecticides derived from the soil bacterium, *Saccharopolyspora spinosa* (Mertz and Yao 1990, Kirst et al. 1992, Yu 2015). Both have the same unique neurotoxic mode of action that involves nicotinic acetylcholine receptors and γ-aminobutyric acid receptors (Shimokawatoko et al. 2012) and have reduced-risk status (EPA 2009). Spinosad is one of several insecticides used in sugar-based baits targeting house flies (Elector, Elanco Animal Health, Greenfield, IN).

Spinetoram was created to be more effective than spinosad (Sparks et al. 2008). It has not yet been formulated against filth flies, although it is used in baits against fly pests on crops (Yee 2018, Baronio et al. 2019). Resistance to spinosad in field populations of house flies has been documented (Markussen and Kristensen 2012, Khan et al. 2013). Cross-resistance with spinetoram has not been examined but might be expected given their same mode of action.

Here, for both experiments, we used female flies that had been given only water between emergence and testing. We used a moderately pyrethroid-resistant strain, which has been reared at the Center for Medical, Agricultural and Veterinary Entomology (CMAVE), United States Department of Agriculture, Agricultural Research Service (Gainesville, FL) since 1958. The susceptible strain was the NIU strain, which is of unknown origin and has been maintained with no exposure to pesticides for >20 yr at Northern Illinois University (DeKalb, IL). The NIU strain is known to be susceptible to permethrin, imidacloprid, and fluralaner and to lack the *kdr* mutations that are associated with resistance to pyrethroids and DDT (unpublished data, Williamson et al. 1993, Burgess and King 2015). For the topical experiment, the flies were tested beginning 0.5–1.5 d after emergence, and they were given 10% sucrose in cotton pads after treatment. For the sugar cube treatment, i.e., oral experiment, the flies were tested beginning up to 1 d after adult sclerotization.

## Experimental Design

The individuals being tested should be as similar as possible, particularly in age, sex, and nutritional state (Yu 2015). When feasible, testing should be with at least three separate batches of insects, each batch being one rearing episode, emergence, or collection date. Within each batch, individual insects should be assigned to treatment at random. Ideally, the batch will be large enough to perform a replicate of each and every treatment (including control). For example, if testing five doses and a control, with 20 flies each, then a batch of at least 120 flies would allow all six treatments to be tested. This is known as a randomized complete block design, although the blocking is not included in the analysis presented here. Batch is the block in this case. A balanced blocked design, such as this, helps minimize any confounding of treatment with batch differences, e.g., in insect quality or in exact testing conditions, such as barometric pressure (Sorzano and Parkinson 2019). If a single batch is too small for a replicate of every treatment, then a balanced incomplete block design may be useful and can be generated in R (GM-RAM 2010, Deagen 2016) or by hand (Sorzano and Parkinson 2019).

## Procedure

### Basic Steps

Using the information in the following sections: Decide what application method to use. Gather materials. Prepare workspace. Dose fix. Prepare solutions. Collect data for probit analysis. Run probit analysis and put findings in table(s). If a goal of your study is to determine relative resistance in relation to a susceptible strain, consult the literature to find a susceptible strain (if one exists) for your species of choice. If an author has recently published data on a susceptible strain, they may be willing to collaborate with you by sending you some to use or to start your own colony.

### Deciding How to Apply Insecticide, e.g., Topically or in Food

- Choose based on other studies, how similar insecticides are used in the field, or ability of the insecticide to penetrate the cuticle.
- Count out and label a suitable number of test containers for the number of doses and controls you plan to run, but do not yet add the flies.

### Materials

**Table:** 

Item (alternatives)	Our source (some alternative(s))	Quantity purchased
Pesticide-grade or ACS-grade acetone	Fishersci.com (sigmaaldrich.com, amazon.com)	1 liter
Dental cotton rolls #2, 38 mm, nonsterile	Amazon.com	2,000 (1 per fly tested)
Sucrose in the form of granulated sugar	Grocery store	≤2.26 kg bag
Water	Water: distilled and/or RO if available (tap water is okay)	
Beta-cyfluthrin (99.5%)	ChemService.com (bocsci.com)	100 mg
Spinosad (98.6% pure analytical standard)	ChemService.com (bocsci.com)	100 mg
Spinetoram (96.4%)	ChemService.com (bocsci.com)	100 mg
PPE (personal protective equipment) chosen from reading SDS for insecticides and acetone: typically, nitrile gloves, lab coat, safety goggles		
Anesthetic: ice (or CO_2_)	Crushed ice machine and freezer (or cryolizer or freeze pack or CO_2_ tank with regulator)	
Pyrex petri dishes (100 mm × 15 mm) for sorting flies by sex and for topical applications	Fishersci.com catalogue # 08-747C	At least 5 per replicate
Wide tip featherweight forceps for moving flies	BioQuip.com catalogue # 4750	At least 2 pairs
Test containers: 150–350 ml (glass jars or disposable ice cream cups; not plastic because acetone solvent may dissolve)	Amazon.com	18 per insecticide if performing 3 replicates of 5 doses or concentrations + a control, plus more for dose fixing if using disposables.
Screening: fiber glass, 1 mm^2^ mesh	Hardware store (department store, amazon.com)	1 roll
Rubber bands (or 3 mm round string elastic cord 23% polyester, 77% rubber)	Department store, amazon.com (fabric store)	At least 2 per container if using rubber bands
1.5-ml calibrated microcentrifuge tubes	Fishersci.com (sigmaaldrich.com, amazon.com)	1 per dose or concentration and control
Single-channel adjustable pipettes ranging from 10 to 1,000 µl		
PB-600 repeating dispenser	Hamilton part no. 83700	At least 2
25-µl point style 3 gastight syringe	Hamilton part no. 80275	At least 2
Sugar cubes	Domino Foods, Inc., Yonkers, NY, ~2.5 g per cube (C & H Brand ~3.5 g per cube)	(1 sugar cube per insecticide concentration tested, plus one for a control) times (the number of replicates)

### Prepare Workspace

- Designate two physically separated areas in the lab, a rearing area and a dose–response area.
- The rearing area should at least be temperature controlled; many species also require a specific humidity range. An environmental chamber that regulates both is ideal, but an alternative is plastic sheeting, a space heater, and a humidifier unit. The dose–response area should be clean with good ventilation.
- Keep clean tools separate from tools that are, or may be, contaminated with insecticide.

### Dose Fix

- Dose fixing is the initial determination of the range of doses or concentrations that will elicit a >0% response at the lowest dose or concentration and <100% at the highest. Yu (2015) suggests five doses ranging from 20% to 80% mortality. However, sometimes, complications in rearing capacity or a desire for tighter control of insect variables like adult age may limit the number of experimental animals available. Here we used three doses in one of the analyses (Table 2, 24 h).
- Pick a starting dose or concentration based on what you find in a literature search of toxicity studies of similar organisms, chemicals, and application methods.
- Plan for doses that cover one order of magnitude above and below the published LD_50_ or LC_50_. If no studies exist, start by using exponentially spaced doses or concentrations, e.g., 0.01, 0.1, 1.0, 10.0, 100.0, 1,000.0. Often, this step requires departing from exponential spacing into something like 5× or 2× spaced doses or concentrations (e.g., dose range in Table 1). Although recommended, the range of doses or concentrations do not have to be proportionately spaced in order to determine LD_50_ or LC_50_ values (e.g., concentration range in Table 3).

**Table 1.** Raw data for response of female house flies of the resistant CMAVE strain and exposed to topical dose of beta-cyfluthrin

Dose (ng insecticide/fly)	Number dead at 24 h (48 h)	Total flies tested
156	100 (91)	100
78	65 (55)	100
39	44 (46)	100
19.5	14 (15)	100
Control	0 (1)	100

**Table 2.** Probit analysis results for female house flies of the resistant CMAVE strain and exposed to topical dose of beta-cyfluthrin

Mortality at	Pooled number of flies for analysis	Slope (SE)	LD_50_ (95% CI) (ng insecticide/fly)	χ ^2^ Goodness-of-fit (*P* value)
24 h	300^*a*^	2.39 (0.33)	50.89a (43.92–60.85)	1.48 (0.22)
48 h	400	2.33 (0.22)	52.17a (11.03–186.63)^*b*^	9.26 (0.01)

LD_50_ values followed by different letters are significantly different from each other based on nonoverlap of the 95% CIs.

The LD_50_ (95% CI) have been corrected for <100% purity of the beta-cyfluthrin, as described in the Analyze Data section. The LC_50_ (95% CI) values prior to this correction, i.e., the values generated by the R code were susceptible strain: 24 h beta-cyfluthrin = 50.64 (43.70–60.55); 48 h beta-cyfluthrin = (51.91 (10.97–185.70).

^*a*^Data from the 100 flies in the highest dose had to be excluded from the 24-h analysis because 100% died.

^*b*^A heterogeneity factor, 4.63 in this example, was used in the calculation of the 95% confidence interval at 48 h because *P* < 0.05 for the χ ^2^ goodness-of-fit test.

**Table 3.** Raw data for response of female house flies of the susceptible NIU strain or resistant CMAVE strain and exposed to either spinosad or spinetoram in the oral experiment

Insecticide	Strain	Concentration (µg insecticide in 3.5-g sugar cube)	Number dead at 24 h	Total flies tested
Spinosad	Susceptible	70	63	80
		35	47	80
		18	38	80
		9	12	80
		Control	0	80
Spinosad	Resistant	70	49	60
		58	46	60
		45	51	60
		33	42	60
		20	28	60
		Control	0	60
Spinetoram	Susceptible	35	68	80
		18	57	80
		9	26	80
		5	8	80
		Control	0	80
Spinetoram	Resistant	100	74	80
		50	65	80
		25	56	80
		13	28	80
		Control	0	80

### Make Solutions

- The solvent typically used is acetone. Acetone has a high vapor pressure and will drip out of standard air displacement pipettes. One workaround is to ‘pre-wet’ the tip with the acetone two to three times before drawing the final volume. Another solution is to use a positive displacement pipette.
- Dissolve 1 mg of insecticide in 1 ml of acetone. In some instances, you may require concentrations higher than this. This is the stock solution. This and subsequent dilutions should be in a nonreactive container, e.g., a polypropylene 1.5-ml microcentrifuge tube or a glass container with a gasket lid. To mix, close the cap and place it on a vortex mixer. Check to be sure all insecticide is dissolved. On rare occasions, some insecticides require a solvent other than acetone, especially at higher concentrations. Making stock solutions within a few hours of when the assays will be conducted is recommended, but longer storage is possible at −20°C in a dark, spark-proof freezer. How long stock solutions can be stored depends on the insecticide.
- Add 500 µl of stock solution to 500 µl of acetone to make a ½ dilution (dilution 1 is 500 µg/ml). For exponential spacing, use 100 µl of stock solution in 900-µl acetone.
- Continue making 1/2 dilutions or exponential dilutions from the previous dilutions until you have at least four dilutions made. For ½ dilutions, dilution 2 is 250 µg/ml, made by adding 500 µl of dilution 1 to 500 µl of acetone; dilution 3 is 125 µg/ml, made by adding 500 µl of dilution 2 to 500 µl of acetone; dilution 4 is 62.50 µg/ml, made by adding 500 µl of dilution 3 to 500 µl of acetone. Dilutions should be stored in tightly sealed containers because acetone readily evaporates, which will change the concentration, and the containers should be stored in a cool, dark place because some insecticides are degraded by light and heat.
- Every time a dose is being tested, also prepare a 1.5-ml microcentrifuge tube with 1 ml of clean acetone as a control.

### Replicate Doses that Will Be Used in Probit Analysis

- Once a range is determined, i.e., the doses are fixed, then begin replicating doses or concentrations within that range. For pesticide registration, EPA requires a minimum of five doses in a dose response. Prior to setting up the bioassays, check whether your government agency or publisher of choice provides any guidelines with regards to recommended number of doses or any other procedural requirements.
- Replicate all doses or concentrations and a control, at least three times or however many times the guidelines recommend. Expect variation in the percent responding for a given dose. For example, two replicates performed a week apart might result in 95% mortality for one replicate and 80% for the other, despite the same dose. This is not unusual.

### Sort Flies

**Fig. 1.**
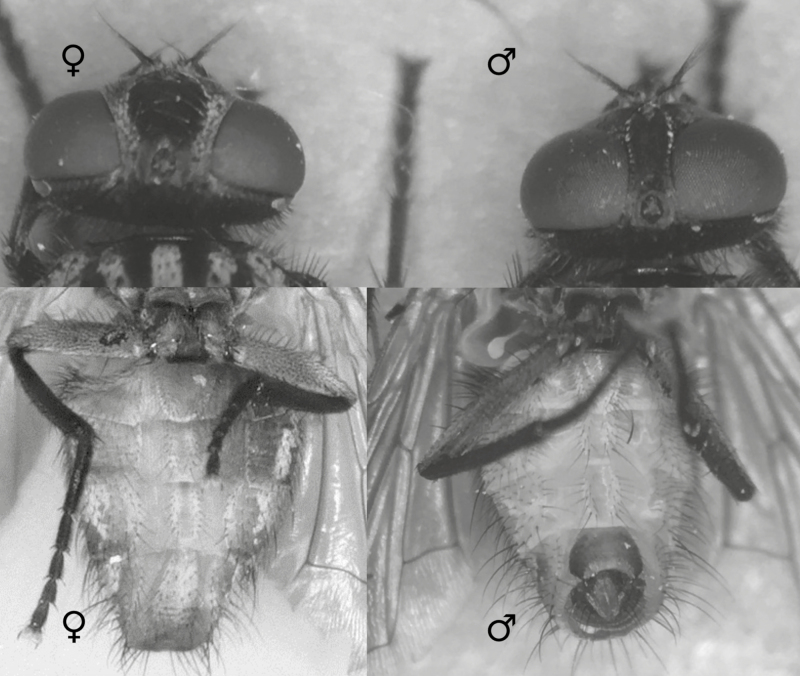
In house flies, *Musca domestica*, unlike females (left), males (right) have dark claspers just anterior to their pigmented copulatory apparatus on their posterior ventral abdomen, and male compound eyes almost touch. Similar sex differences occur in many dipterans.

- Anesthetize flies with cold or CO_2_, e.g., by putting a plastic cage of them in a freezer just until they can all be knocked to the cage floor and show minimal movement. Closely monitor this; staying cold for too long will kill the flies. Then transfer flies to plastic petri dishes buried in a tub of crushed ice for subsequent sorting.
- For each block (See Experimental Design), sort out 20 females (Fig. 1) for each of the doses or concentrations and the control. For 5 doses and a control, you will need 6 × 20 = 120 females.

### To Apply Insecticide Topically

- Take a Pyrex petri dish of 20 anesthetized female flies and apply a single 0.5 µl droplet of your lowest dose being tested to the dorsal thorax of each fly. Do not use plastic petri dishes for this step because acetone will compromise the plastic. A 25 µl Hamilton syringe in a Hamilton PB-600 repeating dispenser will output a 0.5-µl droplet. In general, a Hamilton PB-600 produces a droplet 1/50th the volume of the syringe in it (e.g., 50-µl syringe = 1-µl droplet, 10-µl syringe = 0.2-µl droplet). To calculate how much insecticide is being applied to the fly, take the micrograms per milliliter of insecticide and multiply by the droplet volume; that will be how much insecticide is applied in nanograms to the fly in the droplet (e.g., 1 µg/ml dose = 1 ng/µl, so 0.5-µl droplet = 0.5 ng insecticide applied to the fly). After drawing up enough volume in the syringe to apply one droplet to each of the 20 flies, discard that draw; then redraw the same volume, and dose the 20 flies in rapid succession. The first draw rinses the syringe with the correct concentration, and the rapid application minimizes acetone evaporation from the barrel of the syringe, which would concentrate the toxin
- After the first dose has been applied to all the flies that will receive it, discard the remainder from the syringe into your labeled waste container. (see Cleaning up) Then clean the syringe in preparation for the next dose to be tested, by drawing up and discarding clean acetone 10 times.
- Draw up the second lowest dose in the manner described for the first dose.
- Once all flies have had insecticide applied, place them inside a test container, and secure a piece of screening over the top.
- To prevent the flies from dehydrating and/or starving, place a cotton roll soaked in a sucrose solution, e.g., 10% (v:v) on the screen top. Place containers with the flies in an environmental chamber. Replace food daily until mortality is assessed.

### To Present Insecticide in Food

- Place one sugar cube on the bottom of each container.
- Apply 500 µl of an insecticide treatment (concentration) or control to each sugar cube, always using a new pipette tip. Because you may be working with very small amounts, it is important to avoid a quantity being affected by any residual in the tip.
- Allow the acetone to evaporate for at least 1 h.
- Add 20 anesthetized, presorted flies to each test container, and secure a piece of screening over the top.
- To prevent the flies from dehydrating, place a water-soaked cotton roll on the screen top. Place containers with the flies in an environmental chamber.

### Measure Mortality Response

- Check for mortality at the predetermined time interval, e.g., 24 and/or 48 h. For particularly slow acting insecticides, mortality may be recorded at 72 h or longer.
- Mortality should be clearly defined. Some insecticides can cause symptoms that make the observer assume death (Scott and Georghiou 1984, White et al. 2007), but flies recover from this state (e.g., Table 1: doses 156 ng/fly and 78 ng/fly between 24 and 48 h). Thus, it may be desirable to define mortality as a total lack of movement even when disturbed, which is the definition used here for the oral experiment house fly data. For the topical experiment, mortality was defined as unable to right themselves if on their back and unable to engage in directed locomotion if right-side-up. Often a definition of mortality can be better defined once you know the mode of action of the insecticide.

### Clean up

- Label waste containers (following your department or agency’s protocol).
- Place insecticide-contaminated disposables for chemical waste pickup.
- Rinse insecticide-contaminated reusable glass and the stainless-steel forceps at least twice with acetone, collecting the runoff in a glass waste jug for collection of chemical waste by your institution; then wash as usual, e.g., in warm soapy water, rinsing well afterward, including with a final distilled water or acetone rinse. Supplies should be dry when used.

### Analyze Data

- The numbers that you will be entering into R code (Supp Material [online only]) or other analysis software are those listed in Tables 1 and 3. For peer-reviewed publications, a table of raw data is not required, but sometimes is useful.
- Remember to exclude from analysis the data from doses or concentrations that, when pooled, have 100% or 0% mortality (e.g., results for 24 h in Table 2 excluded the 156 ng insecticide/fly mortalities in Table 1). Including them can interfere with the relationship between the response and dose or concentration being sigmoidal, which is an assumption of the probit analysis. The R code in Supp Material (online only) does not make this exclusion automatically.
- For analysis in R, you can use the code provided in Supp Material (online only).
- If your output shows that the chi-square goodness-of-fit test failed at α = 0.05, i.e., *P* < 0.05, then our R code incorporates a heterogeneity factor into the computation of the CIs (Finney 1971).
- When mortality is >5% in the control, then our R code uses Abbott’s (1925) correction to adjust for deaths in the control (WHO 2018).
- Our R code does not correct for insecticides that are of < 100% purity. To correct, take the LD_50_ or LC_50_ generated by the R code and divide by the percent purity (e.g., LD_50_ = 150 ng/fly and the insecticide is 98.0% purity; 150/0.98 = 153.06 ng/fly when corrected for purity). Whether a purity correction and/or either of the previous two corrections was made should be specified in the methods.
- For analysis in other software, there is free online documentation on how to run probit analyses in POLO, SAS, SPSS, and Prism, to name a few.
- For examples of how to report results, see Tables 2 and 4. Include 1) the total pooled number of individuals tested across all replicates at each dose or concentration, but excluding any control, doses, or concentrations that, when pooled, had 100% or 0% mortality; 2) the slope and SE of the slope; 3) the LD_50_ or LC_50_ values along with their CIs; and 4) the chi-square goodness-of-fit test statistics and associated *P* values.
- Nonoverlapping 95% CIs between different treatments, e.g., different strains or insecticides or formulations, mean that the treatments are significantly different at *P* < 0.05 (e.g., Table 2). The reverse, concluding a lack of statistical difference when 95% CIs overlap, is sometimes done (e.g., Liu and Yue 2000; Burgess and King 2015, 2016), but is statistically conservative, with a nominal α = 0.05, but a real value closer to 0.005, making it harder to detect a significant difference (Wheeler et al. 2006). For some relatively simple alternatives, see Payton et al. (2003) and Wheeler et al. (2006). R code for the ratio test of Wheeler et al. (2006) is provided in Package ‘ecotox’ (Hlina 2020).

**Table 4.** Probit analysis results for female house flies of the susceptible NIU strain or resistant CMAVE strain and exposed to either spinosad or spinetoram in the oral experiment

Insecticide	Strain	Pooled number of flies for analysis	Slope (SE)	LC_50_ (95% CI)^*a*^	χ ^2^ goodness-of-fit (*P* value)
				µg insecticide/g sugar	
Spinosad	Susceptible	320	1.91 (0.24)	7.31b (6.09–8.78)	3.78 (0.15)
Spinosad	Resistant	300	1.83 (0.41)	5.53ab (3.01–7.26)	5.47 (0.14)
Spinetoram	Susceptible	320	2.82 (0.28)	3.89a (3.41–4.43)	2.57 (0.28)
Spinetoram	Resistant	320	2.03 (0.26)	5.17a (3.97–6.28)	3.07 (0.22)

LC_50_ values followed by different letters are significantly different from each other based on nonoverlap of the 95% CIs.

^*a*^The LC_50_ (95% CI) have been corrected for <100% purity of the spinosad and spinetoram, as described in the Analyze Data section above. The LC_50_ (95% CI) values generated by our R code, i.e., prior to the purity correction and before dividing by the sugar mass (3.5 g), were as follows: susceptible strain with spinosad = 25.22 (21.01–30.29), with spinetoram = 13.11 (11.50–14.95); resistant strain with spinosad = 19.07 (10.39–25.06), with spinetoram = 17.45 (13.40–21.19).

### Troubleshoot

- Replicating an assay multiple times will be required before you get a feel for how much variation to expect. If you see variation that seems excessive, sources of error to check for include pipetting technique, calibration of the pipettes, and contaminated solvent. If no errors are found, then the natural variation in the experimental system may be high. If this is the case, five or more replicates of each dose or concentration and control is advisable.
- Do not remove data just because of a lack of statistical fit to a probit regression. If the *P* value of any chi-square goodness-of-fit test is less than 0.05, then the log-transformed data are not adequately explained by the line of best fit, and our R code produces a heterogeneity factor if necessary that is incorporated into the model and given as part of the output (Finney 1971). However, before reporting these results, double check that any control, dose, or concentration that had a pooled 100% or 0% mortality was not the cause of the heterogeneity, i.e., check that they were excluded from the analysis. An example of this is in the topical dose–response results for 48 h in Table 2. Note that the CIs are much wider than they are at 24 h despite there being a similar LD_50_.•Heterogeneity in the model can suggest a mixture of susceptible and resistant individuals in the population being assessed (e.g., Scott et al. 1988). One explanation for a mixture is that the sampled population is really a mix of populations with different insecticide histories and thus different levels of susceptibility to the pesticide. Another explanation is that experimental parameters were not tightly controlled, e.g., that a variable that affects susceptibility was not consistent among replicates or doses or concentrations, e.g., temperature (Yee 2018) or tested individuals’ age, size (Lavadinho 1975), or sex (Ruiu et al. 2007).•If your R output includes any NaNs, this means ‘Not a Number’. Double check that any control, dose, or concentration that had a pooled 100% or 0% mortality was not the cause. Any 100% or 0% mortality will throw off probit analysis.

## House Fly Results

The present study suggests that the LD_50_ values for beta-cyfluthrin are similar between the 24- and 48-h observations. This is not surprising because pyrethroids are known for their quick action.

Spinetoram was approximately two times as toxic as spinosad in the susceptible strain, a significant difference based on nonoverlap of their LC_50_ CIs. This suggests that development of spinetoram-based formulations against house flies may be worthwhile. However, spinetoram will probably not be more toxic than spinosad for all populations. For the pyrethroid-resistant strain, spinosad, and spinetoram, LC_50_ values were very similar. For both spinosad and spinetoram, LC_50_ values were similar between the pyrethroid-resistant and the susceptible strains. Spinosad generally shows little cross-resistance in house flies (Scott 1998, Kristensen and Jespersen 2004, Khan et al. 2014).

Spinosad is considered somewhat slow acting for house flies (Scott 1998). Future studies might address whether spinetoram acts faster than spinosad, by conducting a time series dose response of each and comparing their LT_50_ values (i.e., the time at which 50% of the test population dies). For probit analysis that generates an LT_50_ value, simply replace the doses or concentrations with the period of time, e.g., 1, 4, 8, 24 h. For the Mediterranean fruit fly *Ceratitis capitata*, spinetoram has greater toxicity than spinosad or there is no significant difference, depending on the formulation and whether LC_50_ or LC_95_ is measured (Baldin et al. 2018). Speed of effectiveness as measured by LT_50_ and LT_95_ is very similar for the two insecticides.



### Variations in Procedures

LD_50_ and LC_50_ are widely used to assess toxicity. Ideally, they can be compared among studies. However, it is important to keep in mind that the specific values for LD_50_ and LC_50_ can be affected by details such as the sex and populations or strains being assessed, the method of insecticide application, the environmental conditions under which insects are kept, the time period at which mortality is assessed, and even how mortality is defined (e.g., Barson 1983, Scott 1998, Deacutis et al. 2006).When LD50 and LC50 are being used to examine insecticide resistances of strains, a resistance ratio (RR) can be generated. This ratio is calculated by dividing the LD_50_ or LC_50_ of the resistant strains with the LD_50_ or LC_50_ of the susceptible strain. The RR then represents how many times (fold) greater the value is, or how resistant the resistant strain is compared with the susceptible strain. What constitutes a ‘large’ or ‘small’ RR depends on the species being evaluated. A recent classification for house flies is very low resistance (RR = 5–10), low resistance (RR =  11–20), moderate resistance (RR = 21–50), high resistance (RR = 51–100), and very high resistance (RR > 100; Abbas and Shad 2015, Shah et al. 2017, Wang et al. 2019). One classification system that has been used with mosquitoes is RR < 5 as susceptible, 5 ≤ RR ≤ 10 as moderately resistant, and RR > 10 as highly resistant (WHO 2016).

There are alternative types of bioassays that may require fewer total flies than generating LD_50_ and LC_50_ values. Percent mortality may be compared among populations or insecticides using just one or a few doses or concentrations. Those doses or concentrations might be known amounts of insecticide residue in the environment or recommended application amounts. In these cases, percent mortality would be compared against a positive control (a dose sure to kill 100% of individuals) and a negative control (a dose expected to kill 0%). The one or few doses or concentrations tested for mortality might also be an LD_50_ or LC_50_ value that has already been established for a susceptible population and is used as a standard (a calibration) to quickly and cheaply test for resistance in other populations. For mosquitoes, the Center for Disease Control and the World Health Organization have lists of single diagnostic concentrations for testing for a specified time period under specified conditions for specific genera (CDC 2012, WHO 2018). The diagnostic value is from susceptible individuals exposed to commonly used treatments. Then percent mortality at the specified time is used to assign the population as being susceptible, resistant, or possibly resistant. Multiples of the diagnostic concentration may also be tested to assess the intensity of any resistance.

Dose–response assays and analyses are much more widely applicable then the examples described so far. When doing bioassays to generate dose–response curves, typically, the stimulus is chemical, e.g., an insecticide. However, the stimulus can be biological (e.g., spore dose: Bharadwaj and Stafford 2012, Hajjar et al. 2015; nematode load: Lacey and Unruh 1998) or physical (e.g., humidity: Navaneethan et al. 2010; UV irradiation: Cohen et al. 1975) or time (Baldin et al. 2018). Likewise, the response can be something other than death. For example, dose response is often used to determine how much a chemical, e.g., a potential insecticide, inhibits a target enzyme (Copeland 2013, Swale et al. 2015). In this case, researchers expect enzyme activity to decrease as dose increases. A concentration that inhibits 50% of the enzyme activity is called an IC_50_. When a behavioral response is measured, e.g., a proboscis extension reflex, then researchers talk about an effective dose, e.g., ED_50_ (Hebbalkar et al. 1992).

Other sources of R code for dose–response analysis, besides ours, include, the R package ‘nplr’, which is described in terms of IC_50_ determination (Commo and Bot 2016). In terms of how probit analysis is run, LD, LC, IC, and ED are interchangeable; so for simplicity, our code talks about LD. For nontraditional or complex types of dose–response analyses, researchers should consider an in-depth R package such as ‘drc’ (Ritz et al. 2015).

## Supplementary Material

ieaa041_suppl_Supplementary_Material
