# Supplementary material for: Oral and Topical Insecticide Response Bioassays and Associated Statistical Analyses Used Commonly in Veterinary and Medical Entomology
Source: J Insect Sci. 2020 Nov 2;20(6):6. doi: 10.1093/jisesa/ieaa041 (PMC7604874; doi:10.1093/jisesa/ieaa041)
Supplement: ieaa041_suppl_Supplementary_Material [file ieaa041_suppl_supplementary_material.docx]

#####R CODE

##########################################################################

#Probit Analysis with Heterogeneity Factor Incorporated as Needed and with Abbott's Correction When Control Mortality >5%, and Associated Graph. This code is modified from Johnson et al. (2013).

##########################################################################

### In terms of how probit analysis is run, LD, LC, IC, and ED are interchangeable. For simplicity, the code in this document talks about LD.

###The code in this document adjusts inputted mortality values based on the methods of Abbott (1925) when control mortality exceeds 5%. The adjusted values that are produced are in agreement with Abbott (1925) and in the correction tables presented in Healy (1952).

###Step 1. Enter your data.

#The values for d, r, n, c, cn in the code that follows are the 24 h data found in Table 1 of Burgess et al. (2020).

d = c(78, 39, 19.5)

#Note that d is the dose or concentration, and the format for d is d = c(highest dose to lowest dose, separated by commas)

r = c(65, 44, 14)

# Note that the format for r is r = c(number responding to highest dose to number responding to lowest non-control dose, separated by commas) There should not be any 0 or 100 present between the parenthesis; any non-control doses that give 0 or 100 response values are excluded from the analysis.

n = c(100, 100, 100)

#Note that the format for n is n = c(number tested with the highest dose to the number tested with the lowest non-control dose, separated by commas). If all the numbers are the same, as here, then an alternative format is n = c(rep(100,4)) I.e., n = c(number of total individuals tested at each dose but excluding controls, number of doses excluding control)).

c = (0)

#Note that the format for c is a single number: c = (total number responding in the control)

cn = (100)

#Note that the format for cn is a single number: cn = (total number tested in the control)

conf.level = (0.95)

#Note that this specifies a 95% confidence interval, but you can replace it with an alternative interval. In Burgess et al. (2020), a 95% confidence interval is used, so is what you should use if you are following along with it.

M = c(50)

#Note that this specifies an LD50, as opposed to, e.g., an LD90 value. You can select any number from 1 to 99. If you want, e.g., an LD50 and an LC90, then use M = c(50, 90)

###Step 2. The code that follows up until Step 3 is called the LD function. It will print an output of the probit analysis, tell you if a heterogeneity factor was incorporated, and tell you if Abbott's correction was applied. Any negative corrected values are excluded from the analysis.

############################################

### LD function

############################################

LD <- function(r, n, d, c, cn, M, conf.level) {

## Set up a number series

p <- seq(1, 99, 1)

##Abbott's correction will be applied if there is 5% or more control mortality observed

Mortpercentage <- data.frame(r,n,c,cn)

Mortpercentage$MortPerc <- 1-(Mortpercentage$r/Mortpercentage$n)

Controlpercentage <- 1-(c/cn)

if(Controlpercentage < 0.95) {r2 <- ((Controlpercentage-Mortpercentage$MortPerc)/Controlpercentage)*Mortpercentage$n } else {r2 <- r}

r3 <- round(r2[r2>0],digits = 0)

n2 <- n[order(match(r3,n))]

d2 <- d[order(match(r3,d))]

## r3=number responding, n2=number treated, d2=dose (untransformed)

mod <- glm(cbind(r3, (n2-r3)) ~ log10(d2), family = binomial(link=probit))

##Apply a heterogeneity correction to the confidence intervals according to Finney 1971 (p.72, eq. 4.27; also called "h")

hetfact=deviance(mod)/df.residual(mod)

het = 1-pchisq(summary(mod)$deviance,summary(mod)$df.residual)

ifelse(het > 0.05,1,hetfact)->het ### Heterogeneity cannot be less than 1

## Extract the slope and intercept

summary <- summary(mod, dispersion=1, cor = F)

intercept <- summary$coefficients[1]

interceptSE <- summary$coefficients[3]

slope <- summary$coefficients[2]

slopeSE <- summary$coefficients[4]

z.value <- summary$coefficients[6]

N <- sum(n2)

## Intercept (alpha)

b0<-intercept

## Slope (beta)

b1<-slope

## Slope variance

vcov = summary(mod)$cov.unscaled

var.b0<-vcov[1,1]

## Intercept variance

var.b1<-vcov[2,2]

## Slope intercept covariance

cov.b0.b1<-vcov[1,2]

## Adjust alpha depending on heterogeneity (Finney, 1971, p. 76)

alpha=1-conf.level

if(het > 1) {talpha <- -qt(alpha/2, df=df.residual(mod))} else {talpha <- -qnorm(alpha/2)}

## Calculate g (Finney, 1971, p 78, eq. 4.36)

## "With almost all good sets of data, g will be substantially smaller than 1.0 and

## seldom greater than 0.4."

g <- het * ((talpha^2 * var.b1)/b1^2)

## Calculate theta.hat for all LD levels based on probits in eta (Robertson et al., 2007, pg.

## 27; or "m" in Finney, 1971, p. 78)

eta = family(mod)$linkfun(p/100) #probit distribution curve

theta.hat <- (eta - b0)/b1

## Calculate correction of fiducial limits according to Fieller method (Finney, 1971, pp. 78-79. eq. 4.35)

const1 <- (g/(1-g))*(theta.hat + cov.b0.b1/var.b1) # const1 <- (g/(1-g))*(theta.hat - cov.b0.b1/var.b1)

const2a <- var.b0 + 2*cov.b0.b1*theta.hat + var.b1*theta.hat^2 - g*(var.b0 - (cov.b0.b1^2/var.b1))

const2 <- talpha/((1-g)*b1) * sqrt(het * (const2a))

## Calculate the confidence intervals LCL=lower, UCL=upper (Finney, 1971, p. 78-79. eq. 4.35)

LCL <- (theta.hat + const1 - const2)

UCL <- (theta.hat + const1 + const2)

## Calculate variance for theta.hat (Robertson et al., 2007, pg. 27)

var.theta.hat <- (1/(theta.hat^2)) * ( var.b0 + 2*cov.b0.b1*theta.hat + var.b1*theta.hat^2 )

## Make a data frame from the data at all the different values

LDtable = data.frame(

"p"= p,

"N"= N,

"LD"=10^theta.hat,

"LCL"=10^LCL,

"UCL"=10^UCL,

"slope"=slope,

"slopeSE"=slopeSE,

"intercept"=intercept,

"interceptSE"=interceptSE,

"z.value"=z.value,

"chisquare"=deviance(mod),

"df"=df.residual(mod),

"pchisq"=1-pchisq(summary(mod)$deviance,summary(mod)$df.residual),

"hetfactor"=het,

"HetFactorAdded"=ifelse(het > 1,"yes","no"),

"AbbottCorrectionAdded"=ifelse(Controlpercentage < 0.95, "yes","no"))

## Select output level

return(LDtable[M,])

}

LD(r, n, d, c, cn, M, conf.level)

############################################

## End of LD function

############################################

############################################

#Interpretation of LD function output

############################################

#Below is an explanation of all the values from the output of the LD function. For more details on how they are supposed to look in publications and what they mean, see Burgess et al. (2020). The very furthest number to the left of the output is the row number from the data frame generated by the above function. This is a normal part of R coding and can be disregarded.

#p = the LD value. Example: 50 is the LD50.

#N = the total number of insects tested in all replicates of all doses or concentrations used in the analysis, excluding the control.

#LD = the LD value in the units entered in the variable d.

#LCL = the lower confidence interval value.

#UCL = the upper confidence interval value.

#slope = the slope of the line equation.

#slopeSE = the standard error of the slope of the line equation.

#intercept = the intercept of the line equation.

#interceptSE = the standard error of the intercept of the line equation.

#z.value = the test statistic for the null hypothesis slope = 0.

#chisquare = the test statistic for the Chi-square goodness-of-fit test.

#df = the degrees of freedom for the Chi-square goodness-of-fit test.

#pchisq = the p-value for the Chi-square goodness-of-fit test.

#hetfactor = the value incorporated into the analysis if the Chi-square goodness-of-fit test p < 0.05.

#HetFactorAdded = a yes or no statement confirming or disconfirming that a heterogeneity factor was incorporated.

#AbbottCorrectionAdded = a yes or no statement confirming that Abbott's Correction was or was not applied to the data.

##################################################

## Plot lines on log-probit plot for model assessment

##################################################

#You will only need to install 'ggplot2' one time. After that, you library(ggplot2) to load the package into R.

install.packages ("ggplot2", dependencies=TRUE)

library(ggplot2)

PlotData=data.frame(r,n,d)

prob=r/n

p=ggplot(PlotData,aes(x=log10(d),y=prob))+

geom_point()+

stat_smooth(method="glm",method.args=list(family="quasibinomial"(link="probit")),se=FALSE)

p

############################################################

## Save the output to a .csv file, which can be opened in, e.g., Excel.

############################################################

Data=LD(r, n, d, c, cn, M, conf.level)

#followed by:

write.csv(Data,"NAMEOFYOURFILE.csv")

###########################################################################

#Works Cited

##########################################################################

#Abbott, W. S. 1925. A method of computing the effectiveness of an insecticide. J. Econ. Entomol. 18: 265 - 267.

#Burgess, E. R., B. H. King, and C. J. Geden. 2020. Oral and topical insecticide response bioassays and associated statistical analyses used commonly in medical and veterinary entomology. J. Insect Sci.

#Finney, D. J. 1971. Probit analysis, 3rd ed. Cambridge University Press, New York, NY.

#Healy, M. J. R. 1952. A table of Abbott's correction for natural mortality. Ann. Appl. Biol. 39: 211 - 212.

#Johnson, R. M., L. Dahlgren, B. D. Siegfried, and M. D. Ellis. 2013. Acaricide, fungicide and drug interactions in honey bees (Apis mellifera). PLoS ONE 8: e54092â€“10.
